# Supplementary material for: Identification of Potential Biomarkers for Anti-PD-1 Therapy in Melanoma by Weighted Correlation Network Analysis
Source: Genes (Basel). 2020 Apr 17;11(4):435. doi: 10.3390/genes11040435 (PMC7230292; doi:10.3390/genes11040435)
Supplement: Supplementary file 1 [file genes-11-00435-s001.zip › Supplementary Materials/Table S1.docx]

**Supplementary Material S1** The top 50 node genes in 11 algorithms

| **Degree** | **EPC** | **MNC** | **DMNC** | **MCC** | **BottleNeck** | **EcCentricity** | **Closeness** | **Radiality** | **Stress** | **Betweenness** |
| --- | --- | --- | --- | --- | --- | --- | --- | --- | --- | --- |
| CASP8 | BIRC3 | CD48 | BIRC3 | PSMB8 | CCNG2 | ARHGAP25 | SP100 | SP110 | TYK2 | FKBP5 |
| PTK2B | IL2RB | GBP2 | IL2RB | RAC2 | PSMB8 | BIRC3 | TRIM22 | SASH3 | NFKB2 | PRKACB |
| PRKCB | PIK3CD | STAT5B | PIK3CD | NFKB1 | PDE4B | BTK | RUNX3 | NMI | GBP1 | PDE4B |
| PTPN6 | TAPBP | GBP1 | TAPBP | IL2RB | DOCK8 | CD3E | CD48 | IL7R | JAK2 | TLE3 |
| SP100 | HLA-E | PTPN6 | HLA-E | LCP1 | PRKCB | CD48 | CASP8 | PSMB8 | EZR | LCP1 |
| HLA-A | TAP1 | TRIM38 | TAP1 | TRIM22 | FOXO1 | CD53 | SELL | IRF8 | STAT5B | CD2 |
| PTPRC | GBP2 | ITGAL | GBP2 | HLA-A | NFKB1 | CD8A | STAT1 | IL2RG | FYN | IKZF1 |
| GBP1 | CYLD | CD53 | CYLD | TYK2 | HLA-A | CSF2RB | HLA-E | CSF2RB | ITGAL | CTBP2 |
| STAT1 | IL4R | IL2RB | IL4R | IRF8 | IKZF1 | CYLD | RAC2 | RAC2 | STAT1 | FYN |
| TRIM22 | SELL | SASH3 | SELL | ARHGAP30 | IL2RG | EVI2B | HCLS1 | TRIM21 | CTBP2 | CD48 |
| HLA-E | TRAF3 | HLA-B | TRAF3 | STAT5B | NCOA3 | IL2RB | HLA-A | TYK2 | SELL | ITPR1 |
| IL2RG | HLA-C | HCLS1 | HLA-C | GBP5 | CD8A | IL2RG | IL2RB | IKZF1 | PTPN6 | NFKB1 |
| GBP5 | CSK | IRF8 | CSK | BTK | DNM2 | IRF1 | ITGAL | CXCL10 | EHD1 | LBR |
| TRIM38 | JAK2 | FYN | JAK2 | CORO1A | LCP1 | IRF8 | NMI | GBP1 | DNM2 | RUNX3 |
| TRAF3 | FYN | HLA-A | FYN | PTK2B | PIK3CD | JAK2 | HLA-C | EZR | GBP5 | CD3E |
| GBP2 | ITGAL | CD3E | ITGAL | EVI2B | STK4 | LCP1 | TRIM21 | HCLS1 | CD8A | CORO1A |
| TRIM21 | TYK2 | HLA-C | TYK2 | CD48 | NUP210 | Name | CORO1A | CD53 | CORO1A | IRF1 |
| FYN | SP100 | TRIM21 | SP100 | SP100 | STAT5B | NFKB2 | IRF8 | BTK | IRF8 | SYNE1 |
| SASH3 | LCP1 | BTK | LCP1 | IKZF1 | IL7R | SASH3 | DOCK8 | GBP5 | LBR | RAB35 |
| RAC2 | TRIM38 | CD8A | TRIM38 | IRF1 | RAC2 | SELL | PTPRC | PTPRC | RAC2 | RAC2 |
| HLA-B | GIMAP4 | TRIM22 | GIMAP4 | CD8A | SELL | STAT5B | CD3E | CD2 | TRIM21 | IL7R |
| NFKB2 | PTPN6 | GBP4 | PTPN6 | CXCL9 | ITGAL | TRAF3 | GBP2 | CXCL9 | PSMB8 | CD8A |
| HCLS1 | HCLS1 | LCP1 | HCLS1 | NFKB2 | CASP8 | TRIM21 | GBP5 | CSK | PTK2B | TRAF3 |
| IRF8 | EVI2B | GBP5 | EVI2B | HLA-E | FYN | ABLIM1 | IL2RG | FYN | IKZF1 | PTPN6 |
| CD48 | CFLAR | IL2RG | CFLAR | JAK2 | IRF1 | ARHGEF3 | IL7R | HLA-A | FOXO1 | TRIM21 |
| JAK2 | HLA-B | IRF1 | HLA-B | TRIM38 | IRF8 | CASP8 | JAK2 | PTPN6 | IL2RG | IRF8 |
| BTK | ARHGAP25 | SELL | ARHGAP25 | TRIM21 | BTK | CD2 | NFKB1 | NFKB1 | IL2RB | EHD1 |
| CD2 | TRIM21 | JAK2 | TRIM21 | CD3E | CXCL10 | CORO1A | CSF2RB | CD8A | IL7R | SELL |
| CD3E | GIMAP6 | NFKB2 | GIMAP6 | CSF2RB | CDKN1B | CTBP2 | CD8A | NFKB2 | HLA-E | CXCL10 |
| CORO1A | SP110 | TYK2 | SP110 | ITGAL | ITPR1 | CYFIP2 | LCP1 | TNFSF13B | CD48 | IL2RG |
| CD8A | IL2RG | HLA-E | IL2RG | HCLS1 | CD48 | DNM2 | TNFSF13B | PTK2B | CD3E | EZR |
| IL2RB | SASH3 | CSF2RB | SASH3 | GBP1 | SYNE1 | DOCK8 | BTK | JAK2 | HLA-A | HLA-A |
| CXCL9 | IRF1 | SP100 | IRF1 | STAT1 | UBE2L6 | EML4 | FOXO1 | IL4R | HLA-B | STK4 |
| Name | Name | CASP8 | Name | Name | JAK2 | FOXO1 | Name | Name | Name | Name |
| HLA-C | CD3E | PTK2B | CD3E | TAP1 | LBR | GIMAP7 | GBP1 | LCP1 | TLE3 | PRKCB |
| LCP1 | FMNL1 | NMI | FMNL1 | PTPRC | MAPKAPK2 | IKZF1 | CXCL9 | RUNX3 | LCP1 | CTNNA1 |
| DOCK8 | IRF8 | RAC2 | IRF8 | CD2 | CTNNA1 | IKZF3 | HLA-B | PRKACB | TRAF3 | PTK2B |
| TNFSF13B | STAT5B | PTPRC | STAT5B | IL7R | CD3E | IL16 | SASH3 | PIK3CD | CSF2RB | HLA-E |
| CSF2RB | HLA-A | CXCL9 | HLA-A | IL4R | EHD1 | IL7R | CD53 | HLA-E | CASP8 | ITGAL |
| IL7R | GBP1 | IKZF1 | GBP1 | PTPN6 | IL2RB | ITPR1 | TYK2 | IRF1 | IRF1 | JAK2 |
| PSMB8 | CD53 | CORO1A | CD53 | SELL | TLE3 | NCOA3 | CXCL10 | CORO1A | PRKCB | CASP8 |
| IKZF1 | TRIM22 | ACTR3 | TRIM22 | HLA-C | PTPN6 | NFKB1 | FYN | IL2RB | NFKB1 | PTPRC |
| IRF1 | GBP4 | STAT1 | GBP4 | HLA-B | STAT1 | PRKACB | IKZF1 | STAT1 | PTPRC | STAT1 |
| CXCL10 | CSF2RB | DOCK8 | CSF2RB | FYN | FKBP5 | PSMB8 | CD2 | CD3E | CXCL10 | IL2RB |
| SELL | ARHGAP30 | NFKB1 | ARHGAP30 | GBP4 | TRIM21 | PTPRC | PTPN6 | SELL | CD2 | STAT5B |
| STAT5B | GBP5 | PSMB8 | GBP5 | GBP2 | PTPRC | RAC2 | STAT5B | STAT5B | BTK | FOXO1 |
| GBP4 | BTK | TNFSF13B | BTK | CXCL10 | EZR | RUNX3 | PIK3CD | DOCK8 | CDKN1B | PSMB8 |
| CD53 | NFKB2 | Name | NFKB2 | CD53 | HLA-E | STAT1 | NFKB2 | CASP8 | DOCK8 | DNM2 |
| NFKB1 | NLRC5 | IL7R | NLRC5 | IL2RG | CORO1A | TNFAIP8 | IRF1 | CD48 | SYNE1 | BTK |
| TYK2 | CD8A | CXCL10 | CD8A | SASH3 | CD53 | TNFSF13B | PSMB8 | ITGAL | ITPR1 | UBE2L6 |

EPC, Edge Percolated Component; MNC, Maximum Neighborhood Component; DMNC, Density of Maximum Neighborhood Component; MCC, Maximal Clique Centrality

| **Degree** | **EPC** | **MNC** | **DMNC** | **MCC** | **BottleNeck** | **EcCentricity** | **Closeness** | **Radiality** | **Stress** | **Betweenness** |
| --- | --- | --- | --- | --- | --- | --- | --- | --- | --- | --- |
| CASP8 | BIRC3 | CD48 | BIRC3 | PSMB8 | CCNG2 | ARHGAP25 | SP100 | SP110 | TYK2 | FKBP5 |
| PTK2B | IL2RB | GBP2 | IL2RB | RAC2 | PSMB8 | BIRC3 | TRIM22 | SASH3 | NFKB2 | PRKACB |
| PRKCB | PIK3CD | STAT5B | PIK3CD | NFKB1 | PDE4B | BTK | RUNX3 | NMI | GBP1 | PDE4B |
| PTPN6 | TAPBP | GBP1 | TAPBP | IL2RB | DOCK8 | CD3E | CD48 | IL7R | JAK2 | TLE3 |
| SP100 | HLA-E | PTPN6 | HLA-E | LCP1 | PRKCB | CD48 | CASP8 | PSMB8 | EZR | LCP1 |
| HLA-A | TAP1 | TRIM38 | TAP1 | TRIM22 | FOXO1 | CD53 | SELL | IRF8 | STAT5B | CD2 |
| PTPRC | GBP2 | ITGAL | GBP2 | HLA-A | NFKB1 | CD8A | STAT1 | IL2RG | FYN | IKZF1 |
| GBP1 | CYLD | CD53 | CYLD | TYK2 | HLA-A | CSF2RB | HLA-E | CSF2RB | ITGAL | CTBP2 |
| STAT1 | IL4R | IL2RB | IL4R | IRF8 | IKZF1 | CYLD | RAC2 | RAC2 | STAT1 | FYN |
| TRIM22 | SELL | SASH3 | SELL | ARHGAP30 | IL2RG | EVI2B | HCLS1 | TRIM21 | CTBP2 | CD48 |
| HLA-E | TRAF3 | HLA-B | TRAF3 | STAT5B | NCOA3 | IL2RB | HLA-A | TYK2 | SELL | ITPR1 |
| IL2RG | HLA-C | HCLS1 | HLA-C | GBP5 | CD8A | IL2RG | IL2RB | IKZF1 | PTPN6 | NFKB1 |
| GBP5 | CSK | IRF8 | CSK | BTK | DNM2 | IRF1 | ITGAL | CXCL10 | EHD1 | LBR |
| TRIM38 | JAK2 | FYN | JAK2 | CORO1A | LCP1 | IRF8 | NMI | GBP1 | DNM2 | RUNX3 |
| TRAF3 | FYN | HLA-A | FYN | PTK2B | PIK3CD | JAK2 | HLA-C | EZR | GBP5 | CD3E |
| GBP2 | ITGAL | CD3E | ITGAL | EVI2B | STK4 | LCP1 | TRIM21 | HCLS1 | CD8A | CORO1A |
| TRIM21 | TYK2 | HLA-C | TYK2 | CD48 | NUP210 | Name | CORO1A | CD53 | CORO1A | IRF1 |
| FYN | SP100 | TRIM21 | SP100 | SP100 | STAT5B | NFKB2 | IRF8 | BTK | IRF8 | SYNE1 |
| SASH3 | LCP1 | BTK | LCP1 | IKZF1 | IL7R | SASH3 | DOCK8 | GBP5 | LBR | RAB35 |
| RAC2 | TRIM38 | CD8A | TRIM38 | IRF1 | RAC2 | SELL | PTPRC | PTPRC | RAC2 | RAC2 |
| HLA-B | GIMAP4 | TRIM22 | GIMAP4 | CD8A | SELL | STAT5B | CD3E | CD2 | TRIM21 | IL7R |
| NFKB2 | PTPN6 | GBP4 | PTPN6 | CXCL9 | ITGAL | TRAF3 | GBP2 | CXCL9 | PSMB8 | CD8A |
| HCLS1 | HCLS1 | LCP1 | HCLS1 | NFKB2 | CASP8 | TRIM21 | GBP5 | CSK | PTK2B | TRAF3 |
| IRF8 | EVI2B | GBP5 | EVI2B | HLA-E | FYN | ABLIM1 | IL2RG | FYN | IKZF1 | PTPN6 |
| CD48 | CFLAR | IL2RG | CFLAR | JAK2 | IRF1 | ARHGEF3 | IL7R | HLA-A | FOXO1 | TRIM21 |
| JAK2 | HLA-B | IRF1 | HLA-B | TRIM38 | IRF8 | CASP8 | JAK2 | PTPN6 | IL2RG | IRF8 |
| BTK | ARHGAP25 | SELL | ARHGAP25 | TRIM21 | BTK | CD2 | NFKB1 | NFKB1 | IL2RB | EHD1 |
| CD2 | TRIM21 | JAK2 | TRIM21 | CD3E | CXCL10 | CORO1A | CSF2RB | CD8A | IL7R | SELL |
| CD3E | GIMAP6 | NFKB2 | GIMAP6 | CSF2RB | CDKN1B | CTBP2 | CD8A | NFKB2 | HLA-E | CXCL10 |
| CORO1A | SP110 | TYK2 | SP110 | ITGAL | ITPR1 | CYFIP2 | LCP1 | TNFSF13B | CD48 | IL2RG |
| CD8A | IL2RG | HLA-E | IL2RG | HCLS1 | CD48 | DNM2 | TNFSF13B | PTK2B | CD3E | EZR |
| IL2RB | SASH3 | CSF2RB | SASH3 | GBP1 | SYNE1 | DOCK8 | BTK | JAK2 | HLA-A | HLA-A |
| CXCL9 | IRF1 | SP100 | IRF1 | STAT1 | UBE2L6 | EML4 | FOXO1 | IL4R | HLA-B | STK4 |
| Name | Name | CASP8 | Name | Name | JAK2 | FOXO1 | Name | Name | Name | Name |
| HLA-C | CD3E | PTK2B | CD3E | TAP1 | LBR | GIMAP7 | GBP1 | LCP1 | TLE3 | PRKCB |
| LCP1 | FMNL1 | NMI | FMNL1 | PTPRC | MAPKAPK2 | IKZF1 | CXCL9 | RUNX3 | LCP1 | CTNNA1 |
| DOCK8 | IRF8 | RAC2 | IRF8 | CD2 | CTNNA1 | IKZF3 | HLA-B | PRKACB | TRAF3 | PTK2B |
| TNFSF13B | STAT5B | PTPRC | STAT5B | IL7R | CD3E | IL16 | SASH3 | PIK3CD | CSF2RB | HLA-E |
| CSF2RB | HLA-A | CXCL9 | HLA-A | IL4R | EHD1 | IL7R | CD53 | HLA-E | CASP8 | ITGAL |
| IL7R | GBP1 | IKZF1 | GBP1 | PTPN6 | IL2RB | ITPR1 | TYK2 | IRF1 | IRF1 | JAK2 |
| PSMB8 | CD53 | CORO1A | CD53 | SELL | TLE3 | NCOA3 | CXCL10 | CORO1A | PRKCB | CASP8 |
| IKZF1 | TRIM22 | ACTR3 | TRIM22 | HLA-C | PTPN6 | NFKB1 | FYN | IL2RB | NFKB1 | PTPRC |
| IRF1 | GBP4 | STAT1 | GBP4 | HLA-B | STAT1 | PRKACB | IKZF1 | STAT1 | PTPRC | STAT1 |
| CXCL10 | CSF2RB | DOCK8 | CSF2RB | FYN | FKBP5 | PSMB8 | CD2 | CD3E | CXCL10 | IL2RB |
| SELL | ARHGAP30 | NFKB1 | ARHGAP30 | GBP4 | TRIM21 | PTPRC | PTPN6 | SELL | CD2 | STAT5B |
| STAT5B | GBP5 | PSMB8 | GBP5 | GBP2 | PTPRC | RAC2 | STAT5B | STAT5B | BTK | FOXO1 |
| GBP4 | BTK | TNFSF13B | BTK | CXCL10 | EZR | RUNX3 | PIK3CD | DOCK8 | CDKN1B | PSMB8 |
| CD53 | NFKB2 | Name | NFKB2 | CD53 | HLA-E | STAT1 | NFKB2 | CASP8 | DOCK8 | DNM2 |
| NFKB1 | NLRC5 | IL7R | NLRC5 | IL2RG | CORO1A | TNFAIP8 | IRF1 | CD48 | SYNE1 | BTK |
| TYK2 | CD8A | CXCL10 | CD8A | SASH3 | CD53 | TNFSF13B | PSMB8 | ITGAL | ITPR1 | UBE2L6 |

**Supplementary Material S1** The top 50 node genes in 11 algorithms

EPC, Edge Percolated Component; MNC, Maximum Neighborhood Component; DMNC, Density of Maximum Neighborhood Component; MCC, Maximal Clique Centrality
